# Associations between body fat distribution, insulin resistance and dyslipidaemia in black and white South African women

**DOI:** 10.5830/CVJA-2015-088

**Published:** 2016

**Authors:** Dheshnie Keswell, Mehreen Tootla, Julia H Goedecke

**Affiliations:** Division of Exercise Science and Sports Medicine, Department of Human Biology, University of Cape Town, Cape Town, South Africa; Division of Exercise Science and Sports Medicine, Department of Human Biology, University of Cape Town, Cape Town, South Africa; Division of Exercise Science and Sports Medicine, Department of Human Biology, University of Cape Town, Cape Town, South Africa; Non-communicable Disease Research Unit, South African Medical Research Council, Cape Town, South Africa

**Keywords:** body fat distribution, ethnicity, cardiometabolic risk, dual X-ray absorptiometry, visceral adipose tissue, subcutaneous adipose tissue

## Abstract

**Aim:**

The aim was to examine differences in body fat distribution between premenopausal black and white South African (SA) women and explore the ethnic-specific associations with cardiometabolic risk.

**Methods:**

Body composition, using dual-energy X-ray absorptiometry (DXA) and computerised tomography, insulin resistance (HOMA-IR) and lipid levels were assessed in 288 black and 197 white premenopausal SA women.

**Results:**

Compared to the white women, black women had less central and more peripheral (lower-body) fat, and lower serum lipid and glucose concentrations, but similar homeostasis models for insulin resistance (HOMA-IR) values. The associations between body fat distribution and HOMA-IR, triglyceride and high-density lipoprotein cholesterol concentrations were similar, while the associations with fasting glucose, total and low-density lipoprotein cholesterol levels differed between black and white women.

**Conclusion:**

Ethnic differences in body fat distribution are associated, in part, with differences in cardiometabolic risk between black and white SA women.

## Aim

Recent studies have shown that non-communicable diseases (NCDs) account for the majority of deaths globally (65.5%), and that 80% of the deaths attributed to NCDs each year are in low-middle- income countries.^1^ Within South Africa (SA), NCDs, such as cardiovascular disease (CVD) and type 2 diabetes (T2D) were the second highest cause of death in 2000.^2^ Obesity, a major risk factor for CVD and T2D,^3^,^4^ is extremely common in SA women, particularly in black women.^5^ However, fat distribution rather than the amount of body fat has been shown to be a greater predictor of CVD and T2D risk factors, such as insulin resistance (IR) and dyslipidaemia.^6^-^8^ There is growing evidence indicating that not all fat stores contribute equally to CVD and T2D risk factors.^9^

Studies in predominantly white populations have shown that central fat mass (FM) [measured as trunk fat on the dual-energy X-ray absorptiometry (DXA) scan] or waist circumference,^9^-^11^ and more specifically visceral adipose tissue (VAT),^12^,^13^ is positively associated with IR and dyslipidaemia. Conversely, lower-body (gluteo-femoral) subcutaneous adipose tissue (SAT) has been shown to be negatively associated with these cardiometabolic risk factors.^9^-^11^,^14^ Some, but not all of these studies have demonstrated that central and lower-body fat have independent effects on metabolic risk.^9^-^11^

The relationship between fat distribution and IR appears to be altered by ethnicity. Studies in the USA and SA have shown that compared to white women, black women have less VAT and more gluteo-femoral FM, but are more insulin resistant.^15^-^19^ Less VAT in the black women may, however, explain their more favourable lipid profile compared to white women.^20^ However, SA studies have been performed in only small samples of women (*n* = 10–15) and focused only on abdominal fat distribution.

To date, most studies that have explored ethnic-specific associations between whole-body fat distribution (central and peripheral) and cardiometabolic risk have been undertaken in the USA or Europe, with no studies to our knowledge, examining the independent associations between central and peripheral fat distribution and cardiometabolic risk in African women. Therefore, the aim of this study was to examine differences in whole-body fat distribution between premenopausal black and white women and to explore the ethnic-specific associations with cardiometabolic risk. We also set out to determine whether central versus peripheral fat were independently associated with cardiometabolic risk, and to examine which body composition variable was most closely associated with cardiometabolic risk in black and white women, taking into account other lifestyle factors that have been shown to alter body composition, such as physical activity, use of contraception, smoking and alcohol consumption.

## Methods

The study population consisted of 288 black and 197 white SA women who were recruited by advertisement and from local church groups, community centres and universities in Cape Town, as previously described.^21^ Inclusion criteria were: age 18–45 years; no known diseases or not taking any medication for metabolic disorders; not currently pregnant, lactating or postmenopausal; and being of SA ancestry.

This study was approved by the Human Research Ethics Committee of the Faculty of Health Science, University of Cape Town. Procedures and risks were explained to participants, all of whom gave written informed consent, prior to participation.

The testing procedures and biochemical analyses have been described previously,^21^ but are described briefly below. A demographic questionnaire^22^ was administered and included measures of socio-economic status, including housing density, family history of T2D and behavioural/lifestyle factors. Contraceptive use was recorded, and women were categorised as using hormonal contraception (oral and injection) or not. Smoking was recorded and women were categorised as current smokers or not. Alcohol consumption in grams/day was also recorded using dietary recall.

Physical activity energy expenditure was characterised using the global physical activity questionnaire (GPAQ).^23^ Moderateto vigorous-intensity physical activity (MVPA) was calculated as minutes of physical activity per week.

Anthropometric measurements of participants were taken, including height, weight in light clothing, waist circumference (at the level of umbilicus) and hip circumference (at the largest gluteal area). Body composition (FM and fat-free mass) was measured using DXA (Discovery-W, Software version 4.40; Hologic). Fat mass index (FMI) was calculated as total body fat (kg)/height (m^2^). DXA-derived measures of body fat distribution included trunk, arm and leg FM.

Trunk FM included the region between the neck (line below the bottom of the jaw) and waist cut-offs (line above the iliac crest), with the lateral boundaries positioned to achieve separation of the upper arm and trunk at the glenoid fossa, and the inclusion of vertical lines on either side of the spine were positioned to exclude the spine. The arms included the region below the line through the glenoid fossa. Vertical lines extending downward from the waist cut-off were positioned to separate thigh from hands, and oblique lines were positioned to pass through the femoral neck and join the central vertical line between the legs, in order to isolate the legs.^24^ A CT scan (Toshiba X-press Helical Scanner; Toshiba Tokyo, Japan) at the level of the L4–L5 vertebrae was used to determine VAT and SAT areas

After an overnight fast (10–12 hours), a blood sample was drawn from the antecubital vein for the subsequent determination of plasma glucose, serum insulin, high-density lipoprotein cholesterol (HDL-C), low-density lipoprotein cholesterol (LDLC), total cholesterol (TC) and triglyceride (TG) concentrations. Plasma glucose concentrations were determined using the glucose oxidase method (YSI 2300 Stat Plus; YSI Life Sciences, Yellow Springs, OH). Serum insulin concentrations were determined by immunochemiluminometric assays using the ADVIA Centaur (Bayer Diagnostics, Tarrytown, NJ). Blood lipids were measured using the Roche modular autoanalyser (Roche Diagnostics GmbH, Mannheim, Germany). LDL-C was calculated using the Friedewald formula.25 HOMA-IR was calculated from fasting glucose and insulin levels (glucose (mmol/l) × insulin (mU/l)/22.5).^26^

## Statistical analysis

Results were analysed using Statistica version 10 (Statsoft Inc, Tulsa, Oklahoma, USA). Results are presented as median and interquartile range (IQR). All skewed variables were normalised by log transformation where required. Ethnic differences in body composition, IR and lipid levels were determined using one-way ANCOVA, adjusting for age. Pearson’s chi-squared was used to determine differences in categorical variables between the black and white women. Partial correlations were used to determine the associations between the various body fat distribution variables and cardiometabolic outcomes in the black and white women, adjusting for age and FMI. FMI was chosen as the covariate because it takes into account both the total body fat and the height of an individual (which differs significantly between black and white women).

Multiple regression analysis was used to determine the independent associations between body fat distribution and IR and serum lipid levels, adjusting for age and FMI. In addition, the effect of ethnicity on these relationships was tested by including ethnicity × body fat distribution interaction term in the model. Backward stepwise regression was used to determine the model that accounted for most of the variance for each cardiometabolic outcome. In each model, trunk FM and leg FM were included in the model, with age, FMI, contraception use, MVPA, alcohol consumption and smoking. The analyses were then repeated including VAT and SAT in the model (due to the smaller sample size).

## Results

The black women were younger than the white women [median (IQR): 22 (22–33) vs 32 (24–39] years, p < 0.01] and consequently, all subsequent analyses were adjusted for age. Black women had higher levels of MVPA compared to white women [335 (90–855) vs 240 (120–480) min/week, p = 0.01], and fewer black women smoked (10.1 vs 17%, p = 0.04), whereas alcohol consumption did not differ between the groups [0 (0–2.8) vs 61 (0.5–14.7), p = 0.95]. There was no significant difference in the proportion of women who used contraceptives (32.0 vs 31.1%, p = 0.74), but more black women used injectable contraceptives (25.7 vs 5.1%, p < 0.01), while more white women used oral contraceptives (26.0 vs 6.3%, p < 0.01).

Ethnic differences in body composition and fat distribution are summarised in Table 1. Black women were significantly shorter, heavier, had a higher body mass index (BMI) and greater FM (absolute and %) compared to white women. Black women had greater absolute measures of trunk, leg and arm FM compared to white women. However, as a percentage of total FM, black women had less trunk FM and more leg FM. Accordingly, the trunk FM/leg FM ratio was greater in white than black women. As a percentage of total body FM, there was no significant difference in arm FM between black and white women. Black women had less abdominal VAT and more SAT and a lower VAT/SAT ratio compared to white women.

**Table 1:** Body composition and body fat distribution of black and white women

**	*n*	*Black women median (IQR)*	*n*	*White women median (IQR)*	*p-value adjusted for age*
Body composition					
Height (m)	288	1.60 (1.56–1.64)	197	1.67 (1.60–1.70)	< 0.001
Weight (kg)	288	80.4 (60.9–96.2)	197	73.9 (62.0–94.1)	0.02
BMI (kg/m^2^)	288	31.7 (23.6–37.2)	197	26.6 (22.4–33.2)	< 0.001
Fat (kg)	288	33.7 (19.4–44.3)	197	26.7 (17.1–40.3)	< 0.001
Fat (%)	288	42.1 (32.7–46.9)	197	36.5 (28.9–43.9)	< 0.001
FMI (kg/m^2^)	288	13.3 (7.6–17)	197	9.9 (6.4–14.5)	< 0.001
Body fat distribution					
Waist (cm)	288	94.8 (77.3–108.6)	197	88 (78–101.5)	< 0.001
Trunk FM (kg)	288	14.1 (7.4–20.6)	197	12.2 (7.4–20.3)	0.01
Trunk FM (% FM)	288	42.1 (36.7–46.7)	197	45.5 (40.7–49.7)	< 0.001
Leg FM (kg)	288	13.7 (9.3–18.01)	197	10.7 (7.5–15.9)	< 0.001
Leg FM (% FM)	288	44.3 (39.5–49.4)	197	41.4 (37.5–45.7)	< 0.001
Trunk FM/leg FM	288	0.95 (0.74–1.2)	197	1.1 (0.90–1.3)	< 0.001
Arm FM (kg)	288	3.8 (1.9–4.9)	197	2.9 (1.8–4.6)	< 0.001
Arm FM (%)	288	10.7 (9.3–12.3)	197	10.8 (9.7–11.9)	0.9
VAT (cm^2^)	222	71 (47–102)	153	80 (60–124)	0.04
SAT (cm,^2^)	220	442 (212–577)	150	297 (169–460)	< 0.001
VAT/SAT	220	0.20 (0.14–0.27)	150	0.31 (0.23–0.42)	< 0.001

Values presented as median and interquartile range (IQR);

p-values for one-way ANCOVA adjusting for age.

BMI, body mass index; FMI, fat mass index; WC, waist circumference; FM, fat mass; VAT, visceral adipose tissue; SAT, subcutaneous adipose tissue.

Cardiometabolic risk factors for black and white women are summarised in Table 2. Although there were no ethnic differences in fasting plasma glucose concentrations, black women had higher fasting insulin concentrations and HOMAIR than white women. However, after adjusting for differences in age and FMI, glucose concentrations were significantly lower in the black compared to the white women, but the differences in fasting serum insulin concentrations and HOMA-IR were no longer significant. Black women had lower TC, TG, HDL-C and LDL-C concentrations than white women, which remained significant after adjusting for age and FMI.

**Table 2:** Cardiometabolic risk factors of black and white women

**	*n*	*Black women median (IQR)*	*n*	*White women median (IQR)*	*p-value adjusted for age*	*p-value adjusted for age and FMI*
Glucose (mmol/l)	280	4.5 (4.2–4.9)	196	4.7 (4.4–4.9)	0.08	< 0.001
Insulin (mU/l)	287	9.8 (5.6–16.6)	197	6.9 (4.6–10.8)	< 0.001	0.27
HOMA-IR	279	2.1 (1.1–3.4)	196	1.5 (1.0–2.2)	< 0.001	0.59
TC (mmol/l)	274	3.9 (3.3–4.5)	197	4.7 (4.1–5.3)	< 0.001	< 0.001
TG (mmol/l)	274	0.7 (0.5–1.0)	197	0.9 (0.6–1.2)	< 0.001	< 0.001
HDL-C (mmol/l)	273	1.2 (1.0–1.6)	197	1.6 (1.4–1.9)	< 0.001	< 0.001
LDL-C (mmol/l)	273	2.2 (1.7–2.8)	197	2.6 (2.1-3.3)	< 0.001	< 0.001

Values presented as median and interquartile range (IQR);

p-values for one-way ANCOVA adjusting for age and age and FMI.

HOMA-IR, homeostasis model for insulin resistance; TC, total cholesterol; TG, triglycerides; HDL-C, high-density lipoprotein cholesterol; LDL-C, low-density lipoprotein cholesterol.

Table 3 shows the partial correlations (adjusted for age and FMI) between body fatness and its distribution and cardiometabolic risk factors for the black and white women individually and combined. In summary, age-adjusted total body fat, as defined by FMI, was positively associated with plasma glucose concentrations, measures of IR (fasting insulin and HOMA-IR), TG and LDL-C concentrations, and negatively associated with HDL-C concentrations in both black and white women. FMI was positively associated with TC concentrations in white women only.

**Table 3:** Correlations between body fatness and its distribution and cardiometabolic risk factors in black and white women and the combined sample

**	**	*Glucose*	*Insulin*	*HOMA-IR*	*TG*	*TC*	*HDL-C*	*LDL-C*
FMI (kg/m^2^)	B	0.26^Δ^	0.53^Δ^	0.54^Δ^	0.23^Δ^	0.035	–0.31^Δ^	0.15*
	W	0.23^Δ^	0.61^Δ^	0.61^Δ^	0.35^Δ^	0.26^Δ^	–0.39^Δ^	0.36^Δ^
	All	0.25^Δ^	0.59^Δ^	0.60^Δ^	0.30^Δ^	0.13^Δ$^	–0.34^Δ^	0.25^Δ$^
Trunk FM (kg)	B	0.34^Δ^	0.30^Δ^	0.34^Δ^	0.30^Δ^	–0.060	–0.23^Δ^	–0.039
	W	0.069	0.29^Δ^	0.29^Δ^	0.099	0.043	–0.18^Δ^	0.12
	All	1.04^Δ$^	1.16^Δ^	1.23^Δ^	0.92^Δ^	–0.09^$^	–0.87^Δ^	0.09^$^
VAT (cm,^2^)	B	0.12	0.24^Δ^	0.27^Δ^	0.078	–0.034	–0.099	0.0011
	W	–0.039	0.21^Δ^	0.19*	0.21*	0.10	–0.25^Δ^	0.17*
	All	0.10^$^	0.33^Δ^	0.33^Δ^	0.20^Δ^	–0.04^$^	–0.28^Δ^	0.05^$^
SAT (cm^2^)	B	0.094	–0.023	0.019	–0.11	–0.039	0.15*	–0.071
	W	–0.25^Δ^	–0.0002	–0.017	–0.016	0.15	0.16	0.079
	All	0.13^$^	0.18	0.20	–0.06	–0.20^$^	–0.06	–0.19^$^
Leg FM (kg)	B	–0.16^Δ^	–0.33^Δ^	–0.34^Δ^	–0.23^Δ^	–0.011	0.15*	–0.015
	W	–0.12	–0.25^Δ^	–0.26^Δ^	–0.16*	–0.097	0.19^Δ^	–0.15*
	All	–0.38^Δ^	–0.67^Δ^	–0.69^Δ^	–0.52^Δ^	–0.09	0.39^Δ^	–0.15

Values are presented as partial correlation coefficients adjusted for age and FMI (except for FMI); ^Δ^p < 0.01 and *p < 0.05. ‘All’ values are presented as beta-coefficients adjusted for age, FMI and ethnicity. ^$^Ethnic × body composition interaction. FMI, fat mass index; VAT, visceral adipose tissue; SAT, subcutaneous adipose tissue; FM, fat mass; HOMA-IR, homeostasis model of insulin resistance; TG, triglycerides; TC, total cholesterol; HDL-C, high-density lipoprotein cholesterol; LDL-C, low-density lipoprotein cholesterol.

In black and white women, increased central FM and reduced lower-body fat correlated with measures of IR (fasting insulin and HOMA-IR). In black women only, greater trunk FM and lower leg FM were associated with increased fasting plasma glucose concentrations. Notably these associations with glucose concentrations and trunk FM were significantly different between black and white women. In white women only, increased abdominal SAT was associated with reduced fasting glucose concentrations, and this association differed significantly between black and white women.

In both black and white women, reduced lower-body FM, characterised by leg FM, was associated with TG concentrations. In the black women, higher trunk FM, and in the white women, higher VAT was associated with TG concentrations. In addition, in both the black and white women, trunk FM was associated with reduced HDL-C concentrations. By contrast, higher leg FM was associated with increased HDL-C concentrations in both the black and white women. In white women only, increased VAT and lower-leg FM were associated with increased LDL-C\ concentrations. There were no associations between arm FM and any metabolic risk factor in both black and white women.

We further explored the confounding effects of various lifestyle factors, including contraceptive use, smoking, physical activity and alcohol consumption on metabolic risk. In summary, in black women, IR [2.3 (1.3–3.7) vs 1.8 (1–3.2) mU/l, p = 0.04] was higher and HDL-C concentrations [1.1 (0.9–1.4) vs 1.3 (1.1– 1.6) mmol/l, p < 0.01] were lower in women on contraception than those not on it. Despite few black women consuming alcohol, consumption was positively associated with serum HDL-C concentrations (r = 0.20, p < 0.05) in black women. In white women, TC [5 (4.5–5.5) vs 4.5 (4.1–5.2) mmol/l, p = 0.01], TG [1 (0.7–1.4) vs 0.8 (0.6–1.2) mmol/l, p < 0.01] and HDL-C [1.7 (1.5–2) vs 1.5 (1.3–1.8) mmol/l, p < 0.01] concentrations were higher in women on contraception than those not on it, while lower MVPA was associated with higher fasting insulin concentrations (r = –0.19, p < 0.05) and HOMA-IR (r = –0.18, p < 0.05).

In separate models for black and white women, we then used backward stepwise regression to determine the factors that accounted for the greatest variance in cardiometabolic risk factors, including the following variables in the initial model: age, FMI, trunk FM and leg FM. Based on the results relating to the covariates described above, we also included the covariates contraceptive use and alcohol consumption into the appropriate models (Table 4). We then repeated the regression analyses including VAT and SAT in the models.

**Table 4:** Multivariate analysis for black and white women, separately

	*Black women*				*White women*		
*Variable*	*B*	*p*	*p-value*	*Variable*	*B*	*p*	*p-value*
*Glucose (mmol/l)*				*Glucose (mmol/l)*			
FMI (kg/m^2^)	–1.11	< 0.01		Age (years)	0.18	0.01	
Trunk FM (kg)	1.47	< 0.01		FMI (kg/m^2^)	0.24	0.00	
	r = 0.45	r^2^ = 0.21	< 0.01		r = 0.34	r^2^ = 0.12	< 0.01
*Insulin (mU/l)*				*Insulin (mU/l)*			
Age (years)	–0.33	< 0.01		Age (years)	–0.25	0.00	
Trunk FM (kg)	1.09	< 0.01		Trunk FM (kg)	1.02	0.00	
Leg FM (kg)	–0.53	< 0.01		Leg FM (kg)	–0.37	0.00	
Contraception	0.11	< 0.01					
	r = 0.64	r2 = 0.40	< 0.01		r = 0.67	r^2^ = 0.45	< 0.01
HOMA-IR				HOMA-IR			
Age (years)	–0.29	< 0.01		Age (years)	–0.21	0.00	
Trunk FM (kg)	1.14	< 0.01		Trunk FM (kg)	1.03	0.00	
Leg FM (kg)	–0.57	< 0.01		Leg FM (kg)	–0.38	0.00	
Contraception	0.11	0.02					
	r = 0.65	r^2^ = 0.42	< 0.01		r = 0.67	r^2^ = 0.45	< 0.01
TG (mmol/l)				TG (mmol/l)			
Age (years)	0.12	0.04		FMI (kg/m^2^)	0.90	0.00	
Trunk FM (kg)	0.84	0.00		Leg FM (kg)	–0.51	0.00	
Leg FM (kg)	–0.59	0.00		Contraception	0.26	0.00	
	r = 0.48	r^2^ = 0.23	< 0.01		r = 0.49	r^2^ = 0.24	< 0.01
HDL-C (mmol/l)				HDL-C (mmol/l)			
Age (years)	0.20	0.07		VAT (cm^2^)	–0.45	0.00	
FMI (kg/m^2^)	–1.20	< 0.00		Contraception	0.18	0.01	
Leg FM (kg)	0.77	0.01					
Contraception	–0.23	0.02					
Alcohol consumption	0.23	0.02					
	r = 0.51	r^2^ = 0.26	< 0.01		r = 0.51	r^2^ = 0.26	< 0.01
TC (mmol/l)				TC (mmol/l)			
Age (years)	0.22	0.0021		Age	0.22	0.00	
FMI (kg/m^2^)	0.38	0.025		FMI (kg/m^2^)	0.29	0.00	
SAT (cm^2^)	–0.36	0.029		Contraception	0.29	0.00	
	r = 0.30	r^2^ = 0.10	< 0.01		r = 0.44	r^2^ = 0.19	< 0.01
LDL-C (mmol/l)				LDL-C (mmol/l)			
Age (years)	0.19	0.0071		Age	0.16	0.02	
FMI (kg/m^2^)	0.46	0.0073		Trunk FM (kg)	0.38	0.00	
SAT (cm^2^)	–0.33	0.049					
	r = 0.33	r^2^ = 0.11	< 0.01		r = 0.46	r^2^ = 0.21	7lt; 0.01

In black women, FMI and trunk FM accounted for 21% of the variance in fasting glucose concentrations, whereas in white women, age and FMI contributed significantly to the model, accounting for only 12% of the variance. In both black and white women, trunk FM and leg FM were independently associated with fasting serum insulin concentrations and HOMA-IR, and together with age, and in the case of black women, contraceptive use accounted for 40–45% of the variance in the models. The addition of VAT and SAT to the models did not contribute independently or significantly to the models for fasting plasma glucose and measures of IR in the black and white women.

For the black women, trunk FM and leg FM were independently associated with TG concentrations, whereas only FMI and leg FM, as well as contraceptive use were associated with TG concentrations in the white women. The addition of VAT and SAT did not contribute significantly to the model in both black and white women. In the black women, HDL-C concentrations were independently associated with age, FMI, leg FM, contraceptive use and alcohol consumption, whereas in white women, only VAT and contraceptive use contributed to the model. Notably, the associations between HDL-C concentrations and contraceptive use were opposite in the black and white women, showing a negative association in black women and a positive association in white women.

The model that explained the greatest variance in TC and LDL-C concentrations in the black women included age, FMI and abdominal SAT, the latter being negatively associated with TC and LDL-C concentrations. This contrasts with the findings for white women, where age, FMI and contraceptive use accounted for the greatest variance in TC concentrations, and age and trunk FM contributed to the model for LDL-C concentrations.

## Discussion

The main findings of this study were that compared to white women, black women had less central and more lower-body fat, and lower fasting glucose and lipid concentrations, but had similar levels of IR. Despite these differences, the associations between body fat distribution and measures of IR, as well as TG and HDL-C concentrations were similar in black and white women. The novel finding of this study was that central and peripheral fat depositions were independently associated with IR in both the black and white women, and with TG concentrations in the black women. By contrast, fasting glucose concentrations were associated with centralisation of body fat in black, but not white women, whereas TC and LDL-C concentrations were associated with centralisation of body fat in white, but not black women.

Black women in this study had more total body fat compared to their white counterparts. This is in accordance with recent national SA prevalence data, which reported that black women had a higher prevalence of obesity than other ethnic groups.^5^ However, when adjusting for total body fat, black women had a greater peripheral distribution of fat, characterised by less central FM and more lower-body FM than their white counterparts. Furthermore, within the abdominal depot, black women had less VAT and more SAT compared to white women, which is commensurate with both SA and American studies.^15^-^19^

Less central FM, and to a lesser extent, more peripheral FM in black women, associated with their lower fasting glucose concentrations, suggesting that accumulation of central FM may play a vital role in determining fasting plasma glucose concentrations, and hence the development of T2D in black women. By contrast, despite the differences in body fat distribution, fasting insulin levels and HOMA-IR values were not significantly different between black and white women. Numerous studies have shown that compared to white women, black women have a higher prevalence of IR and T2D for the same BMI or waist circumference.^17^,^19^ These results are surprising, given that greater central and reduced peripheral FM were similarly associated with higher fasting insulin and HOMAIR values in both black and white women, a finding supported by similar studies in the USA.^15^,^27^ These findings suggest that other factors, in addition to body fat distribution, are important determinants of IR in black women.

Another important finding of this study was that central and peripheral FM were independently associated with fasting insulin and HOMA-IR values in both the black and white women. To our knowledge, this is the first study to demonstrate independent associations among ethnically diverse women, a finding that has been demonstrated in mostly white men and women.^9^-^11^ Differences in the contribution of abdominal and gluteo-femoral fat to IR may be due to phenotypic differences in adipose tissue depots. Indeed, studies from our laboratory and others have shown that in white women, inflammatory gene expression, especially in the abdominal depot, was significantly associated with higher IR.^28^-^30^ However, despite black women having a higher SAT inflammatory gene expression profile than white women, SAT inflammatory gene expression was not significantly associated with IR in black women.^28^

In contrast to abdominal fat, lower-body fat has been suggested to act as a metabolic sink, storing excess free fatty acids (FFA) when there is an energy surplus, due to its lower lipolytic activity and higher lipoprotein lipase (LPL) activity, compared to upper-body fat stores.^31^-^34^ Lower-body fat has been suggested to protect against ectopic fat deposition and therefore protect against risk for CVD and T2D. However, when the capacity to store excess fat in the periphery is exceeded, peripheral fat is no longer protective. A small SA study demonstrated reduced adipogenesis and lipogenesis in obese black women, and this was associated with increased IR.^18^ Furthermore, a recent study from our laboratory has shown that with increasing weight gain, black women accumulated more central relative to peripheral FM, which was associated with the development of IR.^35^ These findings imply that the prevention of an increase in centralisation of body fat is vital for the prevention of metabolic risk in black women, and it is important to determine the point at which peripheral FM is no longer protective.

In addition to body fat distribution, other lifestyle factors also influenced IR, but these differed between black and white women. In black women only, contraceptive use, which was predominantly in the form of injectable contraceptives, was associated with increased IR, which is supported by previous studies.^36^ In addition, physical activity was differentially associated with IR in the black and white women. Despite white women having lower MVPA than black women, MVPA was associated with IR in white women only. Previous research from our laboratory has demonstrated that white women mainly perform leisure activity, typically undertaken at a higher intensity, whereas black women mainly perform physical activity for travel, typically undertaken at a lower intensity.^17^,^37^ This may suggest that the intensity of exercise is an important determinant of IR.

HDL-C and TG concentrations are often used as markers for IR.^38^,^39^ Despite similar levels of IR, HDL-C and TG concentrations were lower in the black compared to the white women. Notably, similar to the findings for IR, higher HDL-C and lower TG concentrations were associated with reduced central and increased peripheral FM, and these associations were similar in black and white women, a finding supported by studies in the USA.^40^,^41^ The lower HDL-C concentrations of black women must therefore be explained by other factors.

HDL-C in black women was associated with alcohol consumption, independent of body fat distribution. Alcohol consumption may raise HDL-C concentrations by increasing the transport rate of the major HDL apolipoproteins Apo-I and Apo-II.^42^ Contraceptive use was another significant determinant of HDL-C levels in both black and white women and TG levels in the white women. Notably, contraceptive use was negatively associated with HDL-C concentrations in the black women and positively associated in the white women, which could be explained by the type of contraception used. Studies have demonstrated that women using injectable contraception had lower HDL-C and TG concentrations compared to those who were on oral contraception.^36^

In our study, we also demonstrated that independent of body fat, TC and LDL-C concentrations were lower in the black compared to the white women, which is in agreement with similar SA studies.^22^,^43^,^44^ Additionally, we demonstrated that increased TC and LDL-C concentrations were associated with increased central FM in white women only. Hosain *et al.*^45^ also demonstrated that the association between central FM and lipid levels was stronger in white compared to black women.

It is important to note that increased trunk FM was significantly associated with increased LDL-C concentrations in the white women, whereas in the black women, abdominal SAT area was negatively associated with LDL-C and TC concentration, suggesting a protective effect of SAT in the black women. There have been a number of studies demonstrating that SAT is protective against IR and increased lipid levels, more specifically TG in women with higher BMI.^46^ Additionally, lipodystrophic loss of SAT results in increased IR and dyslipidaemia.^47^

By contrast, other studies in women have demonstrated that loss of abdominal SAT did not produce the same beneficial results as VAT in terms of reduced IR and dyslipidaemia.^48^ This is the first study of which we are aware, that has demonstrated a protective effect of abdominal SAT on cardiometabolic risk in black women. A possible mechanism for the protective role of SAT is that it is an alternative depot for excess FFA, potentially reducing ectopic fat deposition in VAT and the liver, thereby preventing lipotoxicity and reducing dyslipidaemia.^49^

Notably, we found no associations between arm FM and any metabolic risk factor in black and white women. A few previous studies have examined the independent effects of arm vs leg FM on cardiometabolic risk, but the results have been contradictory.^11^,^50^ Although arm FM is regarded as ‘peripheral fat mass’, this is from the upper body and therefore may not exhibit the same protective effects as leg FM. Further studies are required to understand these disparate findings.

The strengths of this study include the state-of-the-art measures of body fat distribution, DXA and CT scans, and the examination of ethnic-specific associations with cardiometabolic risk. Possible limitations of the study were the inclusion of a convenient sample of women, which was not representative of the total population. The black women were more obese that the white women but this may be reflective of the population, according to a recent population survey.^5^ The cross-sectional design of the study limits one to derive conclusions in terms of causality. The number of women in which CT scans were conducted was low (76% of total sample) and this may have created type II error. Furthermore, we did not measure other lifestyle factors such as diet, and this has been shown to affect body fat and cardiometabolic risk. More objective measures of physical activity, using accelerometers, should be used, as this may provide further insight into possible differences in non-exercise thermogenesis within these popul tions. Future studies should also include subjects with a wider age range, as cardiometabolic risk factors differ with age.

## Conclusion

This study showed that black women had lower central and greater peripheral fat compared to white women, which was associated with lower fasting glucose concentrations in the black women and higher TC and LDL-C concentrations in the white women. Increased central and reduced peripheral FM were independently associated with measures of IR in both the black and white women. In addition to body fat distribution, modifiable risk factors were identified, including MVPA, which were associated with reduced IR in the white women, and contraceptive use, which was associated with IR and lipid levels in the black and white women. Intervention studies aimed at reducing centralisation of body fat, increasing physical activity and changing contraceptive use are required to verify these findings in order to provide evidence-based guidelines for the prevention and management of cardiometabolic risk.
